# Functional and Structural Roles of the Major Facilitator Superfamily Bacterial Multidrug Efflux Pumps

**DOI:** 10.3390/microorganisms8020266

**Published:** 2020-02-16

**Authors:** Sanath Kumar, Manjusha Lekshmi, Ammini Parvathi, Manisha Ojha, Nicholas Wenzel, Manuel F. Varela

**Affiliations:** 1 Post-Harvest Technology, ICAR-Central Institute of Fisheries Education, Seven Bungalows, Andheri (W), Mumbai 400016, India; sanathkumar@cife.edu.in (S.K.); manjusha@cife.edu.in (M.L.); 2 CSIR-National Institute Oceanography, Regional Centre, Kochi 682018, India; parvathi@nio.org; 3 Eastern New Mexico University, Department of Biology, Portales, NM 88130, USA; Manisha.Ojha@enmu.edu (M.O.); Nicholas.Wenzel@enmu.edu (N.W.)

**Keywords:** antimicrobial agents, multidrug resistance, bacteria, pathogens, major facilitator superfamily, transporters, sequence motifs, infection

## Abstract

Pathogenic microorganisms that are multidrug-resistant can pose severe clinical and public health concerns. In particular, bacterial multidrug efflux transporters of the major facilitator superfamily constitute a notable group of drug resistance mechanisms primarily because multidrug-resistant pathogens can become refractory to antimicrobial agents, thus resulting in potentially untreatable bacterial infections. The major facilitator superfamily is composed of thousands of solute transporters that are related in terms of their phylogenetic relationships, primary amino acid sequences, two- and three-dimensional structures, modes of energization (passive and secondary active), and in their mechanisms of solute and ion translocation across the membrane. The major facilitator superfamily is also composed of numerous families and sub-families of homologous transporters that are conserved across all living taxa, from bacteria to humans. Members of this superfamily share several classes of highly conserved amino acid sequence motifs that play essential mechanistic roles during transport. The structural and functional importance of multidrug efflux pumps that belong to the major facilitator family and that are harbored by Gram-negative and -positive bacterial pathogens are considered here.

## 1. Introduction

Certain microorganisms, such as bacteria, are causative agents of infection [1]. Furthermore, bacterial pathogens that are resistant to multiple chemotherapeutic antimicrobial agents can constitute serious public health concerns as such microorganisms are potentially untreatable [2,3]. In the clinical healthcare settings, both morbidity and mortality rates of bacterial infections are increasing with time [4]. Among the many virulence factors harbored by bacterial pathogens, those with intrinsic and acquired multidrug resistance determinants not only are refractory to chemotherapy but may also serve as potential targets of modulators [5]. Thus, it is essential to understand at the molecular level the structure-function relationships of bacterial resistance mechanisms [6]. 

In particular, multidrug efflux pumps serve to provide active resistance to a constellation of otherwise potentially useful antimicrobial agents by actively exporting the agents from the intracellular location of bacteria to the extracellular milieu, where such agents are ineffective [7]. Several large superfamilies of transporters contain many thousands of constituents, many of which are devoted to the extrusion of anti-bacterial drugs [8]. The respective members of each of these superfamilies share related amino acid sequences, secondary and tertiary structures, and even specific modes of energetics that drive their efflux activities [9]. Thus, the individual members within their particular superfamily share common ancestral origins and, therefore, evolutionarily conserved sequence motifs, which may serve in mechanistic fashions their active efflux properties [10]. In this review, we consider these sorts of matters as they pertain to the major facilitator superfamily [11,12]. We also discuss specific key bacterial pathogens and address their relevance briefly from a clinical standpoint. 

## 2. Bacterial Pathogens

The major facilitator superfamily is one of the largest secondary transporter families found on Earth, consisting of sub-families harboring homologous and related solute-specific transport systems [11,13]. Multidrug efflux pumps that are members of the major facilitator superfamily can contribute to drug resistance in many pathogens [14]. The global emergence of multidrug-resistant bacteria is increasingly limiting the effectiveness of current drugs and significantly causing treatment failures [15]. Recently, the World Health Organization (WHO) issued a list of priority antibiotic-resistant pathogens of 12 families that hold the greatest threats to human health [16]. These families are divided into three categories according to the urgency of new antibiotics: critical, high, and medium priority. Some of the most severe groups include multidrug-resistant bacteria such as *Acinetobacter*, *Pseudomonas*, and several species from the Enterobacteriaceae family, which are potentially hazardous in hospitals, healthcare facilities, and in the community. Such bacterial pathogens can cause serious bloodstream infections and pneumonia [17]. 

*Klebsiella pneumoniae*, one of the members of the Enterobacteriaceae family, accumulates antibiotic-resistant genes by *de novo* mutations and emerges as a multidrug-resistant and as an extremely drug-resistant (XDR) pathogen [18]. *K. pneumoniae* and *Escherichia coli* are resistant to many antimicrobial agents, including the carbapenems, aminoglycosides, fluoroquinolones, and third-generation cephalosporins [19]. These bacteria account for about one-third of total Gram-negative bacterial infections such as cystitis, pneumoniae, urinary tract infections, endocarditis, and septicemia [20]. *Mycobacterium tuberculosis* strains have acquired intrinsic resistance to multiple antibiotics, limiting the availability of antibiotics for their control [21]. Drug-resistant tuberculosis is one of the significant public health problems that is threatening progress made in its care [22].

Among the multiple drug-resistant pathogens listed is the bacterium *Staphylococcus aureus,* a common cause of nosocomial infections [2,23]. This pathogen causes toxic shock syndrome, endocarditis, septicemia, meningitis, bacteremia, and pneumonia in humans, and many other infectious diseases in cow, buffalo, and sheep, creating severe economic loss [24]. Some common drug-resistant bacteria are also responsible for causing diseases, such as food poisoning by *Salmonella enterica* [25,26], gonorrhea by *Neisseria gonorrhoeae* [27], meningitis by *Neisseria meningitidis* [28], and pneumonia, cardiovascular disease, and acute respiratory disease by *Chlamydia* spp. [29]. Thus, an understanding at the molecular level regarding multidrug-resistant pathogens, their pathogenicity, and control methods may help in new drug discovery and improve their impacts on human as well as non-human animal health. 

## 3. Bacterial Resistance to Antimicrobials 

Bacterial resistance to antimicrobial agents is one of the biggest threats to global public health [30]. The selection of single-drug resistance frequently results in the concomitant selection of multidrug-resistant bacteria, making infections more difficult to treat clinically, leading to alarming numbers of morbidity and mortality associated with these types of microbial pathogens [31,32]. Although antibiotic resistance does develop naturally through evolutionary mechanisms of selective pressure [33], the stifled antibiotics pipeline and misuse of these agents have caused a significant acceleration in the occurrence of antibiotic-resistant infections [34]. Antibiotics were employed as ‘wonder drugs’ to kill microbes, yet decades after the global age of antibiotics during the 20^th^ century, their novel production has nearly halted [34,35]. Thus, new strategies for circumventing bacterial antimicrobial resistance are needed [36]. 

### Mechanisms of Bacterial Resistance to Antimicrobial Agents

Several common biochemical mechanisms utilized by bacteria allow them to tolerate otherwise lethal doses of antibiotics, and it is these mechanisms that ultimately confer a resistant phenotype [5,33,37]. One such common mechanism is the alteration of a drug′s intended target, which most often occurs when bacteria mutate a target protein causing it to become less susceptible to the antimicrobial agent [31,36,38]. Expression of a mutated drug target can spread via transferable genetic elements, such as, for example, plasmids or transposons, to entirely distinct bacterial species [33,39,40]. Another well-known mechanism of resistance involves the inactivation of the antimicrobial agent, which can occur via chemical modification to the drug (as in the case of aminoglycosides) or via lytic processes that cause a drug to be broken down (as in the case of β-lactams) [41,42]. Both inactivation mechanisms have been found on plasmids within drug-resistant strains [43]. 

One of the better-researched mechanisms of bacterial resistance to antimicrobials involves the prevention of a drug from accessing its target via drug-specific efflux pumps [44,45]. The active efflux of antibiotics outside of the bacterial cell lowers the intracellular concentration of drugs, thus promoting survival of the organism and further accretion of mutations within [37]. Efflux pump proteins can be found in the vast majority of known bacterial species, and they are capable of expelling a variety of structurally different drugs, which is attained by taking advantage of an ion-based electrochemical gradient across the membrane or by ATP hydrolysis during antimicrobial transport [37,45,46]. Like the other commonly found mechanisms of resistance, efflux pumps can be encoded on mobile plasmid-borne genetic determinants [45,47]. Over-expression of genes that encode antimicrobial efflux pumps has been linked to an increasing amount of clinically prominent multidrug-resistant pathogens [45]. Bacterial efflux pumps have been organized into five families or superfamilies, discussed below, and reviewed elsewhere [48].

## 4. Transporter Superfamilies 

The bacterial membrane is a semipermeable barrier between the protoplasm and the cell wall, which regulates the movement of substances across the cell. Since bacteria lack membrane-bound organelles to perform processes such as respiration, energy synthesis, and extrusion of toxic substances, the cell membrane assumes versatile roles of performing these functions. Particularly essential for the functioning of the cells are the membrane-bound transport proteins, which perform the critical functions of moving essential nutrients for metabolism and energy generation, and also removing the end products of metabolism to the exterior [49]. Transport proteins also perform an essential function of moving ions in and out of the cell, which is essential for maintaining a difference of electric charge between the inside and outside of the cell membrane. This difference in polarity drives several cellular processes, including the transportation of nutrients and solutes across the membrane [50].

The movement of macromolecules and solutes across the membrane occurs by several mechanisms, which include simple diffusion, facilitated diffusion, and passive and active transport mechanisms. In simple diffusion, the molecules move across the membrane through a phospholipid bilayer from a higher concentration to a lower concentration (downhill) with no expenditure of energy [51]. Gases such as oxygen, carbon dioxide, and macromolecules such as lipids get transported by a simple diffusion process. The facilitated diffusion is similar to simple diffusion, except that the diffusion of hydrophilic molecules occurs through specialized channels [52]. Accordingly, the facilitated diffusion is classified as channel- and carrier-mediated types. In the channel type, the solutes diffuse through protein channels called porins. This mechanism helps to shield the polar and hydrophilic molecules from the hydrophobic core of the plasma membrane [49]. Some of the early and well-characterized porins include the trimeric proteins OmpF, OmpC, and PhoE of *E. coli* [53]. Porin proteins are predominantly β-barrel proteins that allow the diffusion of proteins of less than 600 Da and are non-specific [53,54]. Certain porins open and close in response to specific stimuli [55]. In a carrier-mediated mechanism, the membrane proteins undergo conformational changes after binding with the solute, thus allowing the passage of the molecule across the membrane. These carrier proteins have one or more specific binding sites for their substrates [56]. Either of these processes is not capable of accumulating substrates against the concentration gradient (uphill) [57].

When the activities of a carrier protein are linked to a source of cellular energy such as an electrochemical gradient or utilization of ATP, the transport process is termed active transport [57]. Active transport systems are capable of accumulating substrates against the concentration gradient. These are of two types, namely primary active transport, which involves direct utilization of chemical, redox, or light as the source of energy, and secondary active transport, which is dependent or driven by the energy derived from the metabolic processes of the cell in the form of differences in the electrochemical gradient across the membrane [58].

The discovery of the proton-linked active transport mechanism by Mitchel in 1961 established that cellular metabolism generates an electrochemical gradient across the membrane, which drives numerous cellular processes, including oxidative phosphorylation, and transport of solutes across the membrane [59]. The proposed model involved the chemiosmotic coupling of substrate transport across the membrane. This elegant study demonstrated that the biological oxidation of substrates results in the formation of a chemical gradient of hydrogen ions (protons) or sodium ions across the membrane, which creates a potential difference or electron motive force [59,60]. Proton- and sodium-coupled transporters are termed as secondary active transport and are classified into symport, antiport, and uniport mechanisms [61].

In symport, two molecules are co-transported in the same direction, one of them being an ion such as a proton (H^+^ ion) or a sodium ion, which moves down the ion concentration gradient. The energy generated in the process enables the transport of the second molecule (e.g., a carbohydrate) uphill or against the concentration gradient. The lactose transport system is one of the earliest and extensively studied carbohydrate/H^+^ secondary active transport systems, and it uses protons generated across the membrane as a result of respiration [58,60,61]. Extensively studied sodium-driven solute transport systems include the bacterial melibiose permease, MelB [62,63], and the human glucose transporter SGLT [64,65,66,67]. The antiport mechanism of solute transport consists of two molecules, a solute plus a proton or a sodium ion, moving in opposite directions, such as in the cases of the NorA and NorM multidrug efflux pumps, respectively [68,69,70]. In uniport, a single solute is transported down the concentration gradient. These transporters represent critical model systems for molecular physiological studies of solute and antimicrobial transport.

The secondary active transporters were the focus of intense research in the 20^th^ century. With the discovery of numerous transporter systems in prokaryotes, the classification of these proteins became a difficult task. Functional analysis and sequence comparisons of transporter proteins suggested a common ancestor and distant relationships among the majority of these proteins [51,71]. Based on the structure-function relationships and the sequence similarities, a transporter classification database (TCDB) was created [72]. The TCDB is a curated database that provides evolutionary and functional information on about 74 families or superfamilies, over a thousand families, and many tens of thousands of individual transport proteins [73]. These solute transporters are broadly classified into i) channels/pores, ii) electrochemical potential-driven transporters, iii) primary active transporters, iv) group translocators, v) transmembrane electron carriers, vi) accessory factors involved in transport, and vii) incompletely characterized transport systems [13,74]. 

**Figure 1:**
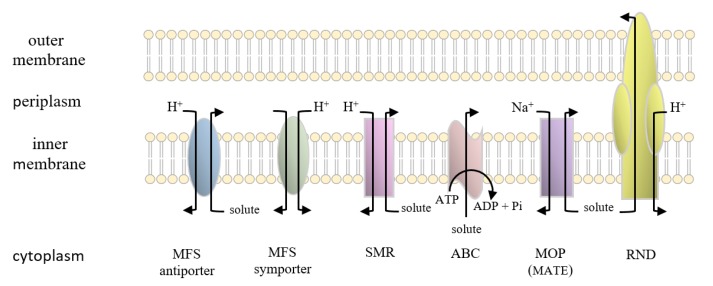
Superfamilies and families of solute transporters. Shown are the outer and inner bacterial membranes with graphic representations of solute transporter superfamilies and families. MFS denotes the major facilitator superfamily; SMR, small multi-drug resistant family; ABC, ATP-binding cassette transporter superfamily; MOP (referred to previously as MATE), multidrug/ oligosaccharidyl-lipid/polysaccharide flippase superfamily; RND, resistance-nodulation-cell division superfamily. H^+^ and Na^+^ denote protons and sodium ions, respectively. Courtesy of Ann Varela.

The secondary active transporters (uniporters, symporters, and antiporters) constitute one of the largest groups in TCDB comprised of four families or superfamilies, viz., a) the major facilitator superfamily (MFS), b) the resistance-nodulation-cell division (RND) superfamily, c) the drug/metabolite transporter (DMT) superfamily, and d) the multidrug/oligosaccharidyl- lipid/polysaccharide (MOP) superfamily [74] (see Figure 1). The RND transporters are known to confer multiple drug resistance and consist of proton-driven tripartite systems, containing, for instance, the AcrB efflux pump in the inner membrane, the AcrA membrane fusion protein in the periplasm, and the TolC protein in the outer membrane [75]. The MOP exporter superfamily harbors several established families of proteins, such as the prokaryotic polysaccharide transporter (PST) family, the bacterial mouse virulence factor (MVF) family, the eukaryotic oligosaccharidyl-lipid flippase (OLF) family, and the multidrug and toxin extrusion (MATE) family of transporters [76,77]. Likewise, the DMT superfamily contains the small multidrug resistance (SMR) family of proteins [78,79,80].

## 5. The Major Facilitator Superfamily 

The major facilitator superfamily of secondary active solute transporters is the second largest group of membrane proteins (Transporter Classification Database, TCDB #2.A.1). The major facilitator superfamily works by symport, antiport, or uniport mechanisms [9,11,81]. These proteins are commonly composed of 400–600 amino acids that fold into 12 or 14 transmembrane helices [82]. The major facilitator superfamily of proteins transport small molecules such as simple sugars, oligosaccharides, amino acids, Krebs cycle intermediates, antibiotics, nucleotides, etc., across the membrane, in both outward and inward directions [13,83]. In order to perform this activity, major facilitator superfamily proteins utilize the ionic (H^+^) gradient across the membrane as the source of energy. This simple, yet elegant mechanism of substrate transport is well conserved across prokaryotic and eukaryotic cellular systems [84,85].

The major facilitator superfamily proteins first discovered by Henderson and colleagues were the sugar symporters [9,84,85]. These investigators demonstrated that sugar transporters from different organisms transporting unrelated substrates were homologous, shared structural similarities, and originated from a common ancestor [9,85]. The sugar transporter proteins transport diverse carbohydrates such as glucose, fructose, mannose, galactose, arabinose, xylose, maltose, lactose, myoinositol, etc., distributed in all three domains, namely bacteria, archaea, and eukarya [86]. A bacterial-specific family of symporters, namely the oligosaccharide-H^+^ symporter (OHS), specifically transport sugars such as lactose, raffinose, and sucrose.

The lactose permease LacY is the earliest sugar/H^+^ symporter to be extensively studied [58,87]. Physiological studies using right-side-out (RSO) membrane vesicles established that the lactose transport is energized by proton-motive force, and the transport of sugar and H^+^ are tightly coupled [87]. Homologous carbohydrate/H^+^ symporters include those that transport metabolically relevant sugars such as D-glucose, D-fructose, D-xylose, L-arabinose, D-galactose, L-rhamnose, L-fucose, and melibiose [46,51]. 

The discovery by Levy and colleagues that homologous membrane proteins, designated as the Tet(A) family of proteins, conferred tetracycline resistance to *E. coli* and revealed that proteins that are structurally related to symporters are involved in the active extrusion of antibiotics from the bacterial cell [44,47,88]. Subsequently, numerous drug/H^+^ antiporters were discovered with considerable structural and functional homologies, although these belonged to diverse bacterial species and transported structurally unrelated substrates [38,89]. 

## 6. Structure-Function Studies of Bacterial Multidrug Efflux Pumps from the Major Facilitator Superfamily 

Several high-resolution crystal structures have been elucidated for well-studied members of the major facilitator superfamily [90,91,92]. A smaller number of these transporters are bacterial multidrug efflux pumps [90]. In a pioneering study, the first multidrug efflux pump of the major facilitator superfamily for which a crystal structure was determined was the EmrD antimicrobial transporter from *E. coli* [93]. Soon afterward, the protein structures for an increasing number of multidrug efflux transporters were elucidated [92]. When considered in light of previous structure-function studies of mutations for many of these solute and antimicrobial transporters, a general structural theme began to emerge. The commonality presumably arises from the prediction that because the members of the major facilitator superfamily share related and conserved sequences, similar two-dimensional structures in the membrane, and similar features in three-dimensional structures, then these transporters possibly share a similar mechanism of substrate transport through the transporters across the membrane [9,84,85]. 

In general, the structures for transporters of the major facilitator superfamily have between 10 and 14 transmembrane α-helices, with the vast majority of these harboring 12 or 14 membrane segments [11]. In addition, the structures have two common types of global bundles. The first bundle type is structural in nature and consists of two mostly symmetrical bundles [9,83,90]. The N-terminal half, the first bundle, is composed of the first six or seven transmembrane α-helices, and the C-terminal bundle consists of the remaining six or seven downstream transmembrane α-helices [11,83]. The second bundle type is functional in its nature and instead is constituted by asymmetries in function [9,84]. In this latter case, it is thought that the two N- and C-terminal bundles have functional differences, accounting for their functional asymmetries, such as dictating specificities in ions and substrates [8]. In evolutionary terms, primordial genetic determinants encoding ancient transporters that likely consisted of six or seven transmembrane segments involving ion conduction, and a gene duplication event early on in the phylogenetic process tandemly attached the duplication to the end of the parental bundle to constitute the C-terminal bundle [8]. Along these lines, it was predicted that the C-terminal bundle then diverged to support the binding and transport of substrates that are increasingly complicated in structure [9,84,94]. In transporters of the major facilitator superfamily, it was observed that water-soluble substrates are accommodated by a sizeable and centrally-located cavity, whereas in antimicrobial efflux pumps the corresponding cavity is somewhat hydrophobic in its property in order to mediate transport of lipophilic antimicrobial agents [93,95,96]. 

**Figure 2:**
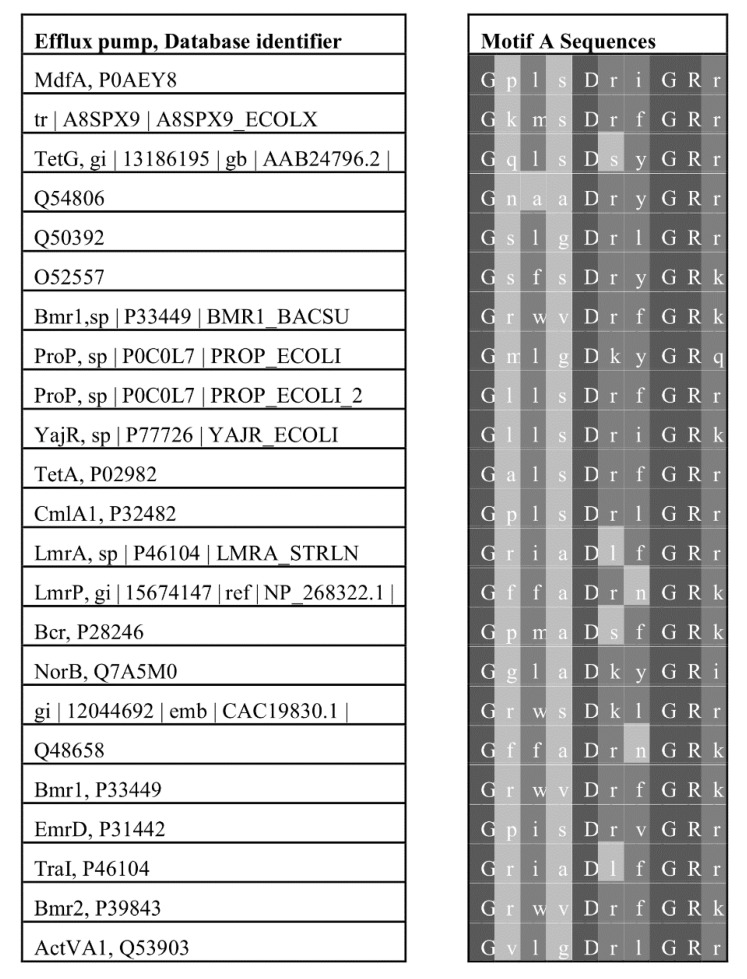
Multiple sequence alignment of motif A of efflux pumps of the major facilitator superfamily. This alignment shows the amino acid residues (right) of the highly conserved motif A of the major facilitator superfamily. This motif lies in the cytoplasmic loop between helices 2 and 3 of the MFS transporters indicated and their respective sequence database identifiers [9,94,98].

Another exciting characteristic of transporters from the major facilitator superfamily is their possession of highly conserved amino acid sequence motifs and their functional relevance [10,97]. One of the earliest known of these conserved motifs, called motif A, consists of residues “G (X)_3_ D R/K X G R R/K” and was discovered by Henderson and colleagues (see Figure 2) [9,94,98].

Motif A resides in a recognizable form within the cytoplasmic loop between transmembrane α-helices two and three of virtually all members of the major facilitator superfamily, including those that are multidrug efflux pumps [83]. Some of the first structure-function analyses of amino acids from motif A were conducted in the laboratories of Levy and Yamaguchi in which they evaluated the dipeptide Ser–Asp of the loop, and since the serine was not conserved, these investigators postulated a requirement of a negatively charged residue for substrate binding and, thus, tetracycline transport [99,100]. When strongly conserved residues of motif A, such as Gly-62, Asp-66, Gly-69, and Arg-70, were evaluated by mutation and transport studies in multidrug efflux pumps, the corresponding residues were postulated to form components of the transport pathway [101,102], a transporter gate [100,103], a stabilizing or mechanistic set of salt-bridges [104] as seen in the LacY symporter [58,105,106], a regulator of conformational changes [101,102], an interface between the C- and N-terminal bundles [104], a device for sensing the electrochemical gradient status [107,108], and a conformational switching mechanism [107]. 

**Figure 3:**
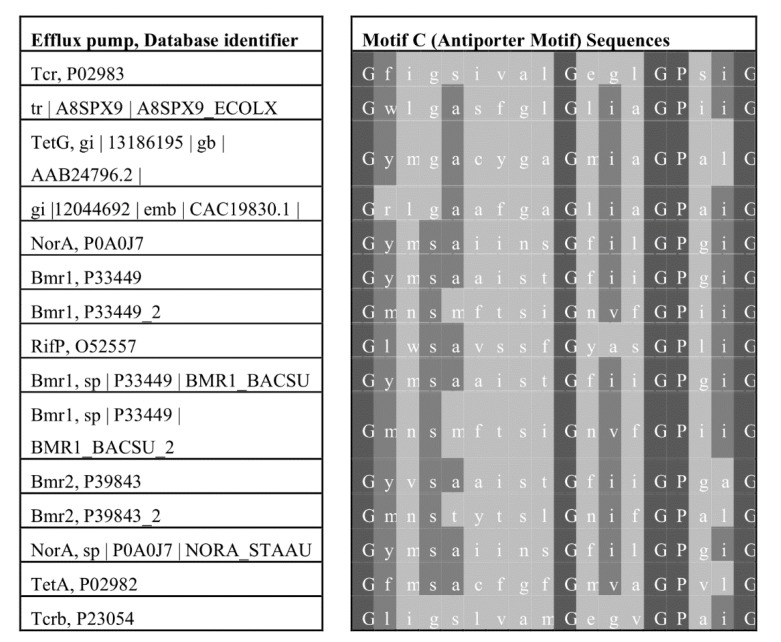
Multiple sequence alignment of motif C in efflux pumps of the major facilitator superfamily. Included in this alignment are highly conserved residues (right) of motif C, also known as the antiporter motif and is located within the middle of helix 5 of transporters from the major facilitator superfamily; included are the transporter protein designations and database identifiers (left) [46,98,110].

A distinctive and highly conserved amino acid sequence motif called motif C [109] and the antiporter motif [110] has been demonstrated to be harbored by drug–ion antiporters of the major facilitator superfamily [98,111]. The general sequence consensus is G (X)_8_ G (X)_3_ G P(X)_2_ G G, and it resides within the fifth α-helix of antiporters of the major facilitator superfamily; see Figure 3 for alignments [98]. If the sequence alignments are manually modified, then elements of the motif appears in most members of the superfamily [112]. The first study to confirm functional importance by systematic mutation was performed by Varela and Griffith on the motif’s most highly conserved residue, Gly-147, of the TetA(C) tetracycline efflux pump [110]. The structural–functional analysis showed that the glycine residue of the motif was necessary for conferring antibiotic resistance, and that the structure formed by the antiporter motif was kinked [110]. These functional and structural features of motif C were confirmed by a number of subsequent investigations that were recently reviewed by Kumar et al. [97]. For instance, various elements of the antiporter motif were postulated to play functional roles in substrate direction of transport [11,84], conformational changes during the drug transport cycle [113], a leakage barrier preventing unwanted ion-substrate coupling [114,115,116], a structure stabilization system [117,118], antimicrobial agent binding [119], a central substrate-binding cavity [120], an interface between the two global bundles [112], a molecular hinge mechanism [121], and a regulator of conformational switching during antimicrobial efflux [121].

## 7. Modulation of Multidrug Efflux Pumps of the Major Facilitator Superfamily 

Due to their widespread occurrence among cells from across all known living taxa and because of their ability to confer multiple antimicrobial resistance, bacterial multidrug efflux pumps from the major facilitator superfamily make suitable targets for resistance modulation [38,89,122]. A variety of efflux pump modulators have been discovered, such as naturally-occurring bioactive agents [123,124], synthetic agents [125], and synergistic modulator combinations [126]. Table 1 lists some examples of various modulators of antimicrobial efflux pumps belonging to the major facilitator superfamily, which are discussed in detail elsewhere [38]. 

One of the earliest clear examples of modulation upon a major facilitator superfamily antimicrobial efflux pump was that of the energy uncoupler carbonyl cyanide *m*-chlorophenylhydrazone (CCCP) and the TetA(C) tetracycline efflux pump [157], demonstrating that the pump was a secondary active transporter. Since this groundbreaking study, CCCP has been used as a means of establishing the ion-driven process of energization for most newly discovered secondary active transport systems [7,158]. Furthermore, CCCP has been shown to be effective, albeit in an indirect manner, as an inhibitor of antimicrobial efflux in a great variety of major facilitator superfamily transporters by collapsing the proton motive force [38,89,122]. Along these lines, reserpine and piperine have served as general inhibitors for many efflux pumps, independent of the mode of energy, substrates, and superfamily membership [159,160,161].

A universal target for a multitude of efflux pump inhibitors is the NorA transporter from the critical pathogen *S. aureus* and is considered in further detail elsewhere [162,163]. Similarly, the QacA efflux pump from *S. aureus* represents another well-studied target for modulation by a large number of inhibitors, which have been extensively reviewed [124,164,165]. In our laboratory, we discovered that the non-toxic cumin spice extract and its bioactive agent cuminaldehyde inhibited resistance and efflux, respectively, which were mediated by the multidrug efflux pump LmrS from *S. aureus* [133,166]. More recently, brachydin-based compounds extracted from extracts of *Arrabidaea brachypoda* were shown to inhibit both the growth of *S. aureus* and NorA drug efflux [156]. As clinical infection by *S. aureus* is a critical public health concern and because the genome encodes over a dozen distinctive antimicrobial efflux pumps, this bacterium will continue to be a target of intensive study for resistance modulation [167,168,169].

We also evaluated the efficacy of the garlic extract and its bioactive agent allyl sulfide towards multidrug resistance conferred by the EmrD-3 multidrug efflux pump from the *Vibrio cholerae* pathogen [128]. We found a direct effect upon antimicrobial transport across EmrD-3 by garlic extract at low concentrations but an indirect effect on resistance at higher garlic extract amounts, probably through modulation at the level of the respiratory chain [128]. Correspondingly, we observed similar modulatory effects with cumin and drug transport through LmrS and with the energetics of the respiratory chain in *S. aureus* [133]. We anticipate that similar direct effects on antimicrobial transport at low modulator concentrations and indirect effects at relatively higher modulator amounts will continue to be observed with other bacterial pathogens that harbor multidrug efflux pumps that constitute members of the major facilitator superfamily.

Previously known as CmlA and Cmr, and now as MdfA, the protein structure of this multidrug efflux pump from *E. coli* was determined at high resolution in which one of its substrates, chloramphenicol, plus two substrate analogs and putative efflux pump inhibitors *n*-dodecyl-*N,N*-dimethylamine-*N*-oxide and deoxycholate, were bound to MdfA [170]. Interestingly, chloramphenicol makes contact with the conserved and negatively-charged residues Glu-26 and Asp-34, which are located in α-helix one of MdfA and are encircled by conserved members of motif C, namely, Val-149, Ala-150, Ala-153, and Pro-154, constituting the so-called domain interface between the two global bundles [170]. In more recent studies, it was discovered that not only is the α-helical structure formed by the motif C kinked, as predicted [110], but the fifth helix also rotationally twists during substrate translocation across the membrane [171]. Thus, because of its presence in efflux pumps of the major facilitator superfamily, it is anticipated that the domain interface component of the molecular hinge is a desirable target for the development of potent efflux pump inhibitors [90].

## 8. Concluding Remarks

Bacterial pathogens are equipped with virulence factors that confer alarming numbers of morbidity and mortality and move through human and animal populations. Predictions of deaths due to multidrug-resistant pathogens are not terribly reassuring [172]. Thus, the study of virulence factors necessitates their continued attention. As such, various strategies are necessary in order to address and combat the further spread of bacterial infection [36]. Bacterial resistance to multiple antimicrobial agents serves as practical virulence factors [173]. Thus, drug and various drug resistance mechanisms make suitable targets for modulation [38,48]. The discovery of new modulators for bacterial multidrug efflux pumps will continue to be an active area of investigation [123]. It is anticipated that prospective studies will be geared towards the clinical application of newfound modulators in synergistic combinations towards infectious bacterial disease. Combination therapy may undoubtedly be of tremendous utility in the unforeseeable future.

Despite intensive investigative efforts aimed towards understanding the various bacterial resistance determinants, the precise molecular pathways and the mechanisms that energetically drive the transport of multiple antimicrobial agents across the membrane through the multidrug efflux pumps remain poorly understood. Much work is needed from a structure-function perspective to elucidate how multidrug transporters invoke passive versus active systems of solute transport across the membrane. Similarly, investigators are still grappling with elucidating the precise molecular mechanisms of energy transduction in both primary and secondary active antimicrobial efflux pumps and in understanding how these transporter systems transfer their respective ATP- and ion gradient-driven energies into actual translocation of substrates through their transporter avenues across the membrane.

Knowledge of protein structures for multidrug efflux pumps may improve our understanding of antimicrobial transport if such integral membrane proteins are evaluated in terms of the drug translocation cycles inherent in the transporters. The antimicrobial transport cycles across the membrane through efflux pumps are currently known in a rudimentary fashion, which makes it challenging to apply towards elucidation of modulator modes of action. It is predicted that by combining studies that address conformational changes during transport with activities of effective inhibitors, our understanding of the mechanisms of new drug actions toward multidrug efflux pump targets will be enhanced.

In addition, it remains unclear how individual bacterial efflux pumps orchestrate substrate specificities. In some cases, an induced fit model has been invoked to explain the antimicrobial specificity profile for specific efflux pumps. In these multidrug efflux pumps, the biochemical contacts that are present within the substrate-binding site are composed of several amino acids that constitute a malleable location in order to accommodate the structurally dissimilar repertoire of antimicrobial agents. Given that many of these microbial efflux pumps are evolutionarily conserved and constitute thousands of individual members in a handful of superfamilies, it is thus unknown how these related antimicrobial transporters nevertheless dictate their different substrate specificity profiles. Along these lines, molecular knowledge is lacking regarding the mechanisms involved in determining whether a given efflux pump accommodates single or multiple substrates.

In addition to the above-mentioned strategies for addressing the serious problem of bacterial multidrug resistance, other avenues are relevant. For instance, from a fundamental standpoint, education of the public is a significant approach, whether through news outlets or social media. Educational institutions can implement local and regional handwashing and personal hygiene programs. Public health policymakers can play relevant roles in these efforts and can significantly aid in reducing the conditions that foster multidrug resistance and transfer between susceptible human populations. These public outreach avenues aside, various strategies can be implemented in scientific circles. For instance, reduction of antimicrobials in clinical and agricultural settings have already confirmed a corresponding decrease in incidences of clinical infection. As an example, the incidence numbers of *Streptococcus pneumoniae* were decreased after reducing the use of antimicrobials [174]. It is anticipated that similar practices will aid in furthering these same sorts of outcomes. Again, public health policy officials can enhance and improve these successes.

Novel antimicrobials with distinctive modes of action are needed. Towards these possibilities, an assortment of approaches have been tried. One of the most promising of these methods involves comparative genomics [175,176,177]. Comparing the whole genomes of known pathogens with those of strains from closely-related non-pathogenic counterparts can reveal key genetic elements that are indispensable for the growth and survival of pathogens and is a consistently promising approach. Such genomics-based comparisons will identify novel targets for the development of novel therapeutics. Essential cellular factors may encode novel targets for antibacterial agents. Such factors include those for metabolism, growth, synthesis of membrane and proteins, energetics, gene expression regulation, and virulence. Furthermore, combining the use of novel modulators against novel bacterial targets with synergistic drug mixtures as a new paradigm is predicted to enhance therapeutic efficacies concerning infection [36].

Another approach towards the discovery of novel antimicrobials is bioinformatics-based. By combining knowledge of structure-function relationships between multidrug resistance mechanisms with active site elucidations, investigators may design new modulators that inhibit these resistance factors. Alternatively, bioinformatics can be applied to the discovery of genetic elements that encode virulence and resistance determinants. Thus, new resistance-conferring determinants can be exploited in order to predict novel structures, mechanisms of action, and new inhibitors that disrupt them.

As one considers the many existing and presently unknown molecular approaches for the new target discovery of novel antibacterial agents, it is apparent that much investigation is needed on many of these fronts. It is our hope that eventually these multivariate avenues of development will inform positive outcomes in diminishing bacterial infection.

## Figures and Tables

**Table 1:** Some examples of various modulators of antimicrobial efflux in some bacterial efflux pumps from the major facilitator superfamily.

Efflux Pump Targeted	Modulators	References
EmrB from *Escherichia coli*	Phenylalanine arginyl β-naphthylamide (PAβN) and 1-(1-naphthyl methyl)-piperazine (NMP)	[127]
EmrD-3 from *Vibrio cholerae*	Garlic, allyl sulfide	[128]
LmrP from *Lactococcus lactis*	Verapamil and quinine Nicardipine and vinblastine Tetraphenyl phosphonium	[129]
QacA from *Staphylococcus aureus*	Hydantoin, silybin	[130,131]
MdfA from *Escherichia coli*	Reserpine	[132]
QacB from *Staphylococcus aureus*	Silybin	[131]
LmrS from *Staphylococcus aureus*	Cumin seed oil, cumin aldehyde, reserpine	[133]
NorA from *Staphylococcus aureus*	3-aryl piperidines	[134]
	Berberine	[135]
	Reserpine	[136]
	Omeprazole, lansoprazole	[137]
	GG918, tariquidar (primary active transport inhibitors)	[138,139]
	Verapamil, ciprofloxacin, ofloxacin	[140]
	5,9′dimethyl-deca-2,4,8-trienoic acid, 9-formyl-5-methyl-deca-2,4,8-trienoic acid	[141]
	Chlorpromazine, thioridazine, and prochlorperazine	[142,143,144]
	Kaempferol rhamnoside	[145]
	Chalones	[146]
	COX-2 inhibitor analog, 3-(4-chlorophenyl)-1-(4-nitrophenyl)-1,4-dihydropyrazolo[4,3-c] [1,2] benzothiazine 5,5-dioxide	[147]
	Coumarin	[148]
	Genistein (flavonoid compound)	[131]
	Ginsenoside 20(S)-Rh2	[149]
	Boronic acid molecules, 6-(3-phenylpropoxy) pyridine-3-boronic acid and 6-(4-phenylbutoxy) pyridine-3- boronic acid	[150]
	Silybin	[151]
	5′-methoxy-hydnocarpin, pheophorbide A, 5′-MHC, curcumin, kaempferol, silibinin, isoflavone, orizabins, capsaicin, tannic acid,	[152]
	nerol, dimethyl octanol, estragole	[153]
	Riparin B	[154]
	Olaanolic acid, ulvaol	[155]
	Brachydins: BR-A, BR-B	[156]
